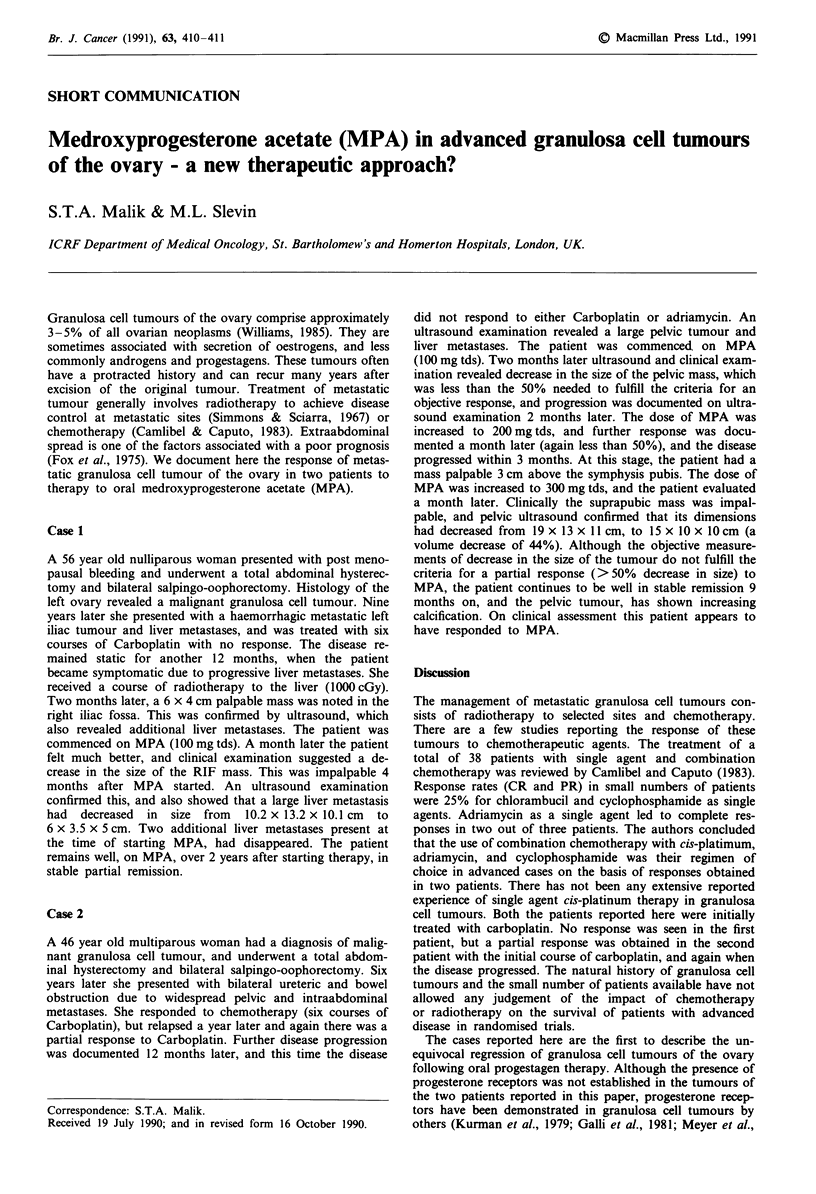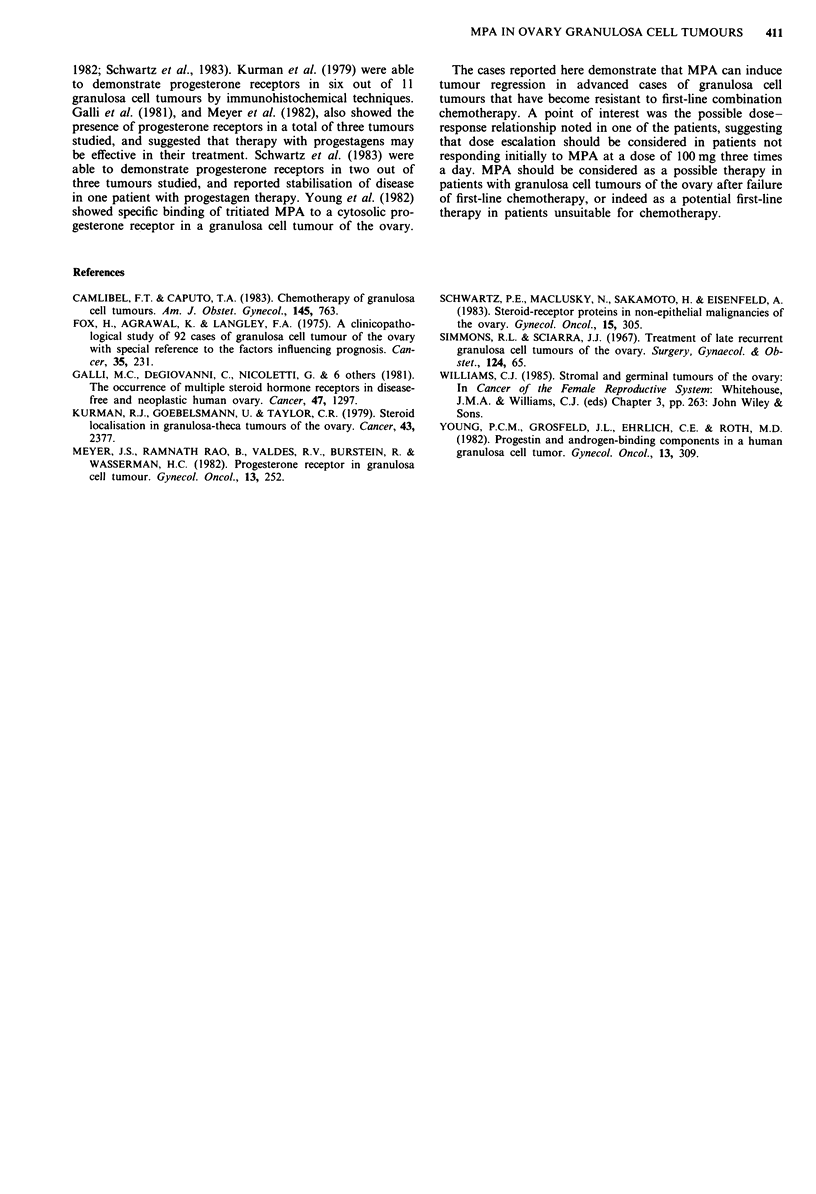# Medroxyprogesterone acetate (MPA) in advanced granulosa cell tumours of the ovary--a new therapeutic approach?

**DOI:** 10.1038/bjc.1991.94

**Published:** 1991-03

**Authors:** S. T. Malik, M. L. Slevin

**Affiliations:** ICRF Department of Medical Oncology, St. Bartholomew's Hospital, London, UK.


Br. J. Cancer (1991), 63, 410-411

© Macmillan Press Ltd., 1991

SHORT COMMUNICATION

### Medroxyprogesterone acetate (MPA) in advanced granulosa cell tumours of the ovary - a new therapeutic approach?

S.T.A. Malik & M.L. Slevin

*ICRF Department of Medical Oncology, St. Bartholomew's and Homerton Hospitals, London, UK.*

Granulosa cell tumours of the ovary comprise approximately 3–5% of all ovarian neoplasms (Williams, 1985). They are sometimes associated with secretion of oestrogens, and less commonly androgens and progestagens. These tumours often have a protracted history and can recur many years after excision of the original tumour. Treatment of metastatic tumour generally involves radiotherapy to achieve disease control at metastatic sites (Simmons & Sciarra, 1967) or chemotherapy (Camlibel & Caputo, 1983). Extraabdominal spread is one of the factors associated with a poor prognosis (Fox *et al.*, 1975). We document here the response of metastatic granulosa cell tumour of the ovary in two patients to therapy to oral medroxyprogesterone acetate (MPA).

### Case 1

A 56 year old nulliparous woman presented with post menopausal bleeding and underwent a total abdominal hysterectomy and bilateral salpingo-oophorectomy. Histology of the left ovary revealed a malignant granulosa cell tumour. Nine years later she presented with a haemorrhagic metastatic left iliac tumour and liver metastases, and was treated with six courses of Carboplatin with no response. The disease remained static for another 12 months, when the patient became symptomatic due to progressive liver metastases. She received a course of radiotherapy to the liver (1000 cGy). Two months later, a 6 × 4 cm palpable mass was noted in the right iliac fossa. This was confirmed by ultrasound, which also revealed additional liver metastases. The patient was commenced on MPA (100 mg tds). A month later the patient felt much better, and clinical examination suggested a decrease in the size of the RIF mass. This was impalpable 4 months after MPA started. An ultrasound examination confirmed this, and also showed that a large liver metastasis had decreased in size from 10.2 × 13.2 × 10.1 cm to 6 × 3.5 × 5 cm. Two additional liver metastases present at the time of starting MPA, had disappeared. The patient remains well, on MPA, over 2 years after starting therapy, in stable partial remission.

### Case 2

A 46 year old multiparous woman had a diagnosis of malignant granulosa cell tumour, and underwent a total abdominal hysterectomy and bilateral salpingo-oophorectomy. Six years later she presented with bilateral ureteric and bowel obstruction due to widespread pelvic and intraabdominal metastases. She responded to chemotherapy (six courses of Carboplatin), but relapsed a year later and again there was a partial response to Carboplatin. Further disease progression was documented 12 months later, and this time the disease did not respond to either Carboplatin or adriamycin. An ultrasound examination revealed a large pelvic tumour and liver metastases. The patient was commenced on MPA (100 mg tds). Two months later ultrasound and clinical examination revealed decrease in the size of the pelvic mass, which was less than the 50% needed to fulfill the criteria for an objective response, and progression was documented on ultrasound examination 2 months later. The dose of MPA was increased to 200 mg tds, and further response was documented a month later (again less than 50%), and the disease progressed within 3 months. At this stage, the patient had a mass palpable 3 cm above the symphysis pubis. The dose of MPA was increased to 300 mg tds, and the patient evaluated a month later. Clinically the suprapubic mass was impalpable, and pelvic ultrasound confirmed that its dimensions had decreased from 19 × 13 × 11 cm, to 15 × 10 × 10 cm (a volume decrease of 44%). Although the objective measurements of decrease in the size of the tumour do not fulfill the criteria for a partial response (> 50% decrease in size) to MPA, the patient continues to be well in stable remission 9 months on, and the pelvic tumour, has shown increasing calcification. On clinical assessment this patient appears to have responded to MPA.

### Discussion

The management of metastatic granulosa cell tumours consists of radiotherapy to selected sites and chemotherapy. There are a few studies reporting the response of these tumours to chemotherapeutic agents. The treatment of a total of 38 patients with single agent and combination chemotherapy was reviewed by Camlibel and Caputo (1983). Response rates (CR and PR) in small numbers of patients were 25% for chlorambucil and cyclophosphamide as single agents. Adriamycin as a single agent led to complete responses in two out of three patients. The authors concluded that the use of combination chemotherapy with *cis*-platimum, adriamycin, and cyclophosphamide was their regimen of choice in advanced cases on the basis of responses obtained in two patients. There has not been any extensive reported experience of single agent *cis*-platinum therapy in granulosa cell tumours. Both the patients reported here were initially treated with carboplatin. No response was seen in the first patient, but a partial response was obtained in the second patient with the initial course of carboplatin, and again when the disease progressed. The natural history of granulosa cell tumours and the small number of patients available have not allowed any judgement of the impact of chemotherapy or radiotherapy on the survival of patients with advanced disease in randomised trials.

The cases reported here are the first to describe the unequivocal regression of granulosa cell tumours of the ovary following oral progestagen therapy. Although the presence of progesterone receptors was not established in the tumours of the two patients reported in this paper, progesterone receptors have been demonstrated in granulosa cell tumours by others (Kurman *et al.*, 1979; Galli *et al.*, 1981; Meyer *et al.*,

Correspondence: S.T.A. Malik.
Received 19 July 1990; and in revised form 16 October 1990.

1982; Schwartz *et al.*, 1983). Kurman *et al.* (1979) were able to demonstrate progesterone receptors in six out of 11 granulosa cell tumours by immunohistochemical techniques. Galli *et al.* (1981), and Meyer *et al.* (1982), also showed the presence of progesterone receptors in a total of three tumours studied, and suggested that therapy with progestagens may be effective in their treatment. Schwartz *et al.* (1983) were able to demonstrate progesterone receptors in two out of three tumours studied, and reported stabilisation of disease in one patient with progestagen therapy. Young *et al.* (1982) showed specific binding of tritiated MPA to a cytosolic progesterone receptor in a granulosa cell tumour of the ovary.

The cases reported here demonstrate that MPA can induce tumour regression in advanced cases of granulosa cell tumours that have become resistant to first-line combination chemotherapy. A point of interest was the possible dose–response relationship noted in one of the patients, suggesting that dose escalation should be considered in patients not responding initially to MPA at a dose of 100 mg three times a day. MPA should be considered as a possible therapy in patients with granulosa cell tumours of the ovary after failure of first-line chemotherapy, or indeed as a potential first-line therapy in patients unsuitable for chemotherapy.

**References**

CAMLIBEL, F.T. & CAPUTO, T.A. (1983). Chemotherapy of granulosa cell tumours. *Am. J. Obstet. Gynecol.*, **145**, 763.

FOX, H., AGRAWAL, K. & LANGLEY, F.A. (1975). A clinicopathological study of 92 cases of granulosa cell tumour of the ovary with special reference to the factors influencing prognosis. *Cancer*, **35**, 231.

GALLI, M.C., DEGIOVANNI, C., NICOLETTI, G. & 6 others (1981). The occurrence of multiple steroid hormone receptors in disease-free and neoplastic human ovary. *Cancer*, **47**, 1297.

KURMAN, R.J., GOEBELSMANN, U. & TAYLOR, C.R. (1979). Steroid localisation in granulosa-theca tumours of the ovary. *Cancer*, **43**, 2377.

MEYER, J.S., RAMNATH RAO, B., VALDES, R.V., BURSTEIN, R. & WASSERMAN, H.C. (1982). Progesterone receptor in granulosa cell tumour. *Gynecol. Oncol.*, **13**, 252.

SCHWARTZ, P.E., MACLUSKY, N., SAKAMOTO, H. & EISENFELD, A. (1983). Steroid-receptor proteins in non-epithelial malignancies of the ovary. *Gynecol. Oncol.*, **15**, 305.

SIMMONS, R.L. & SCIARRA, J.J. (1967). Treatment of late recurrent granulosa cell tumours of the ovary. *Surgery, Gynaecol. & Obstet.*, **124**, 65.

WILLIAMS, C.J. (1985). Stromal and germinal tumours of the ovary: In *Cancer of the Female Reproductive System*: Whitehouse, J.M.A. & Williams, C.J. (eds) Chapter 3, pp. 263: John Wiley & Sons.

YOUNG, P.C.M., GROSFELD, J.L., EHRLICH, C.E. & ROTH, M.D. (1982). Progestin and androgen-binding components in a human granulosa cell tumor. *Gynecol. Oncol.*, **13**, 309.